# Outcomes of a Program to Support Outpatient Antibiotic Adherence Among People Experiencing Housing Instability With Serious Injection Drug-Related Infections in Boston, MA: A Propensity Score Matched Study

**DOI:** 10.1093/ofid/ofag481

**Published:** 2026-08-05

**Authors:** Nicole C McCann, Elizabeth Glaser, Jake R Morgan, Dominic Hodgkin, Ghulam Karim Khan, Simeon D Kimmel

**Affiliations:** Department of Health Law, Policy, and Management, Boston University School of Public Health, Boston, Massachusetts, USA; Boston Healthcare for the Homeless Program, Boston, Massachusetts, USA; Department of Health Law, Policy, and Management, Boston University School of Public Health, Boston, Massachusetts, USA; Institute for Behavioral Health, Heller School for Social Policy and Management, Brandeis University, Waltham, Massachusetts, USA; Boston Healthcare for the Homeless Program, Boston, Massachusetts, USA; Section of Infectious Diseases, Boston Medical Center, Boston, Massachusetts, USA; Boston University Chobanian & Avedisian School of Medicine, Boston, Massachusetts, USA; Section of Infectious Diseases, Boston Medical Center, Boston, Massachusetts, USA; Boston University Chobanian & Avedisian School of Medicine, Boston, Massachusetts, USA; Section of General Internal Medicine, Boston Medical Center, Boston, Massachusetts, USA

**Keywords:** homelessness, housing instability, injection drug-related infections, outpatient support, people who inject drugs

## Abstract

**Background:**

Antibiotics are effective for treating serious injection drug-related infections (SIRIs). However, completion rates are often low among people who inject drugs. We evaluated Boston Healthcare for the Homeless Program Infectious Disease Outreach Liaison Team (BOLT), an outpatient liaison program for people experiencing housing instability hospitalized with SIRI, intended to increase intravenous or oral antibiotic completion.

**Method:**

We included BOLT enrollees (1 June 2023 to 31 August 2024) and historical controls (1 June 2020 to 28 February 2023). Primary outcomes were the number of re-hospitalizations and emergency department (ED) visits in the 90-day period following the SIRI index hospitalization. The secondary outcome was probability of patient-directed discharge (PDD) from the index hospitalization. BOLT enrollees and historical controls were matched on demographic and clinical characteristics. In regression analyses, we further adjusted for observed characteristics in a doubly robust approach.

**Results:**

Prior to matching, BOLT enrollees and historical controls were significantly different on most characteristics, with BOLT enrollees having higher prevalence of osteomyelitis and epidural abscess SIRIs, and opioid and stimulant use disorders. The matched analytic sample included 74 BOLT enrollees and 121 historical controls. The matching approach achieved balance on most characteristics except osteomyelitis prevalence and methadone receipt. In regression analyses, 90-day re-hospitalization was similar between BOLT enrollees and controls (*P* = .982). BOLT enrollees had an adjusted mean of 0.35 fewer ED visits per enrollee versus controls over the 90-day postindex hospitalization period (*P* = .288). Probability of PDD was similar between groups (*P* = .946).

**Conclusions:**

BOLT was not significantly associated with changes in acute health care utilization. This may reflect unobserved differences between BOLT enrollees and historical controls and suggests a need for additional research to understand program implementation and more proximal health outcomes.

## Teaser

Boston Healthcare for the Homeless Program Infectious Disease Outreach Liaison Team, a novel outpatient liaison program to improve antibiotic completion in people experiencing housing instability hospitalized with serious injection drug-related infections, was not associated with changes in hospitalization or emergency department visits in the 90-day postintervention versus matched historical controls.

Alongside the US opioid overdose crisis, hospitalizations from serious injection-related infections (SIRIs) have more than doubled since the early 2000s [1, 2]. Serious injection-related infections are significant contributors to morbidity and mortality among people who inject drugs (PWIDs) [2–4]. Antibiotics are effective for treating SIRIs; however, antibiotic completion rates are often low in PWID. A Boston-based study found that among patients hospitalized with injection-related endocarditis, only 65% completed antibiotics, with 30% leaving the hospital or postacute care prior to completion [5].

Historically, SIRIs have been treated with intravenous (IV) antibiotics, often requiring prolonged hospitalization or skilled nursing facility admission to complete treatment. Although IV antibiotics can be administered at home via long-term peripherally inserted catheters with visiting nurse support, and research suggests this is safe for some PWID, PWIDs have often been excluded from these programs due to unstable housing or concerns about risk of catheter infection or manipulation [6, 7]. However, growing evidence suggests that oral antibiotic therapy for certain SIRIs may be safe and effective, particularly for patients with patient-directed discharge (PDD) who are unable to complete prolonged IV antibiotics [8–11]. A systematic review found that PWIDs prefer outpatient management of SIRI when possible [10]. Data also suggest that multidisciplinary outpatient supports are needed for PWID with PDD on oral antibiotics [11]. Due to challenges with traditional treatment strategies, people experiencing housing instability with SIRIs may be more likely to receive oral antibiotics yet face additional barriers to adherence and may need specialized supports to improve outcomes.

The Boston Healthcare for the Homeless Program (BHCHP) Infectious Disease Outreach Liaison Team (BOLT), first implemented in June 2023, is a novel intervention involving a partnership between colocated health systems, BHCHP (a federally qualified health center with outpatient and respite services), and a single hospital, Boston Medical Center (BMC), the largest safety-net hospital in New England, serving a high proportion of patients with substance use disorder (SUD) and experiencing housing-related needs. BOLT was designed by clinicians at BHCHP and BMC to meet this patient population's needs and was funded by the Massachusetts Department of Public Health. BOLT aims to improve antibiotic completion in people experiencing housing instability hospitalized with SIRIs. The intervention involves a coordinated 3-person team consisting of an infectious disease physician, nurse, and case manager to identify patients with SIRIs experiencing housing instability (homelessness or unstable housing) in Boston at risk for PDD during hospitalization and to provide supportive care and ongoing outreach. Patients are referred to the program through automated chart review, proactive outreach, or consult orders and are enrolled while in-hospital. BOLT coordinates with the patient's primary clinical team, infectious disease service, and addiction medicine service when applicable, and conducts regular check-ins to promote adherence. Prior to discharge, BOLT assesses outpatient needs, for example, whether the patient needs access to a cell phone, linkage to primary care, addiction treatment, additional infectious disease treatment (eg, human immunodeficiency virus [HIV], Hepatitis C), housing resources, or case management. Coordination and follow-up occur regardless of discharge method; if PDD occurs, BOLT follows patients to support outpatient antibiotic completion and other needs. This intervention was implemented amidst growing evidence that (1) oral antibiotics can be effective for selected serious infections and (2) among PWID who leave PDD, receipt of oral antibiotics is associated with better outcomes than no antibiotics and similar outcomes as PWID who receive IV antibiotics in-hospital [8, 12, 13]. There has been increased emphasis on shared decision-making in antibiotic selection for people with SIRIs, yet it is not known if these changes are associated with changes in clinical outcomes.

The objective of this analysis was to evaluate whether BOLT was associated with lower 90-day re-hospitalization, 90-day emergency department (ED) utilization, and PDD among BOLT enrollees compared with matched historical controls. We hypothesized that BOLT will be associated with reductions in these outcomes.

## METHODS

### Data Sources

We used data from the BMC Clinical Data Warehouse for Research, which contains electronic health record (EHR) information for BMC patients from June 2019 through December 2024. The EHR data contain patient-reported demographics, health system encounters, diagnostic codes, medications administered during hospitalization and at discharge, clinical orders, and discharge disposition information from within the BMC health system [14]. We also used BOLT enrollment data, which contain patient information and their enrollment date, allowing linkage to the EHR. The Boston University Medical Campus Institutional Review Board approved the study.

### Sample

The treated sample included individuals enrolled in BOLT in the first 15 months of the program, from 1 June 2023 to 31 August 2024. We considered their index hospitalization to be the hospitalization documented in the EHR overlapping with their BOLT enrollment date. Multiple hospitalizations could overlap with the BOLT enrollment date if a patient was discharged and readmitted on the same day. In the few cases where this happened, we conducted chart review to determine the correct matching hospitalization (eg, the longer hospitalization). Historical controls were drawn from a sample of individuals hospitalized with SIRIs at BMC from 1 June 2020 to 28 February 2023. To define SIRIs, we used International Classification of Diseases-10th edition (ICD-10) codes for endocarditis, septic arthritis, epidural abscess, and bacteremia. We also used ICD-10 codes to identify qualifying SUDs (Supplementary Table 1). Contemporaneous controls were not used because BOLT is an operational program which aims to enroll all eligible individuals, so we were concerned that using unenrolled individuals during the time in which BOLT was available would result in selection bias. We defined inclusion criteria for the historical control group to match BOLT enrollment criteria as closely as possible based on clinical expertise. To be included in the control group, individuals had to meet the following criteria: (1) be hospitalized with a SIRI for >24 hours; (2) have no SIRI hospitalization in the 3 months before index hospitalization; (3) have an SUD diagnosis in the 3 months before or during hospitalization, including opioid, cocaine, or other stimulant SUD based on ICD-10 codes (Supplementary Table 1); and (4) have a past-year history of housing instability (including homelessness or unstable housing) based on a validated algorithm that aggregates EHR-based structured data to identify homelessness and housing instability [15]. We required controls to be hospitalized for at least 24 hours to improve matching, as patients could only be enrolled in BOLT if they remained in the hospital long enough to be identified and approached. For individuals with their first hospitalization in the first 3 included months (June through August 2020), we used a lookback period of 3 months and excluded anyone who had a SIRI-related hospitalization between March and May 2020. Historical controls’ first hospitalization in the included period was considered their index hospitalization.

For both BOLT enrollees and comparators, we excluded individuals discharged to hospice or to law enforcement, and those who died during the index hospitalization.

### Outcomes

Primary outcomes were inpatient utilization and ED utilization in the 90 days after discharge from the index SIRI hospitalization. Inpatient and ED utilization were defined using encounter data. Inpatient utilization included hospitalizations and observation unit stays. If an ED encounter resulted in a linked hospital encounter, the encounter was considered a hospitalization. Both inpatient and ED utilization were defined as the per-patient count of encounters in the 90 days following discharge from the index hospitalization. A secondary outcome was PDD, a binary variable based on discharge disposition data. We selected outcomes that were meaningful clinically and to the health system and that were measurable in available data sources.

### Sample Characteristics: Sociodemographic and Clinical Variables

We included sociodemographic variables available in the EHR: age, gender, race, ethnicity, and insurance category. Patient clinical characteristics at the time of index hospitalization included: age-adjusted Charlson comorbidity score [16], SIRI and SUD diagnoses, and past 30-day inpatient and ED utilization. We also included index hospitalization details, including length of stay, whether it included intensive care unit (ICU)–level care, whether SUD medication treatment was administered during the hospitalization (ie, buprenorphine, methadone, naltrexone, or off-label use of acamprosate or topiramate for alcohol and stimulant use disorder), and whether consults were ordered (addiction, infectious disease, psychiatry, or surgery), and selected discharge medications: medication for opioid use disorder (MOUD), oral antibiotics, IV antibiotics, and medications potentially implicated in polypharmacy (medications that may negatively interact with illicit substances among PWID [eg, clonidine, gabapentin, see Supplementary Table 2] [17]).

### Analysis

We first described sample characteristics in the control and BOLT groups and tested for differences between groups. We reported unadjusted primary and secondary outcomes, using χ^2^ tests for categorical variables and Wilcoxon rank-sum tests for continuous variables.

In our main comparative analysis, we used a propensity score matching approach [18]. We generated propensity scores using a probit model of study group membership, based on the following explanatory variables: age, gender, race, ethnicity, insurance category, comorbidity score, SIRI type, SUD category, past 30-day inpatient and ED utilization, whether the index hospitalization was associated with an ICU stay, whether MOUD was administered during the index hospitalization, and whether there was a prescription for a polypharmacy-related medication at discharge (Supplementary Table 2). For SIRI type, we created mutually exclusive categories in which patients were assigned to the most severe SIRI experienced during the index hospitalization. Severity was determined based on typical clinical course and duration of treatment. Endocarditis was considered the most severe, followed by epidural abscess, septic arthritis, osteomyelitis, and then bacteremia/unspecified (unspecified only in cases of BOLT enrollees without SIRI ICD-10 codes). The variables used in matching and their definitions were selected as variables that could affect selection into BOLT, but were not BOLT outcomes, based on input from clinical experts and the BOLT team.

After generating propensity scores, we conducted matching using a greedy nearest neighbor approach with a caliper of 0.2 [19]. This approach pairs each enrollee with controls that have the closest propensity scores within the caliper to prevent poor-quality matches, while allowing for more flexibility than exact matching, which would have left many enrollees unmatched, given differences between groups and our small sample. We used 1:3 matching to maximize power given our small treated sample size. We imposed common support trimming, excluding BOLT observations whose propensity score was higher than the maximum or less than the minimum of the controls. We ran ordinary least squares regression comparing BOLT enrollees and matched historical controls. We further adjusted for the variables used for matching in regression, resulting in a doubly robust approach.

In sensitivity analyses, we varied matching approaches by using: 1:2 matching, 1:1 matching, matching without replacement, matching without imposing common support, varying the caliper (from 0.1 to 0.3), and using an inverse probability weighting approach. We also varied our regression specifications, using logistic regression for the binary PDD outcome and negative binomial regression for the inpatient and ED utilization outcomes (counts). We further evaluated utilization outcomes as binary probabilities (ie, yes/no over the 90-day postindex hospitalization period), rather than continuous counts.

All analyses were conducted in STATA Version 17.0.

## RESULTS

### Sample Characteristics

Of 76 total BOLT enrollees, 1 was excluded due to discharge to hospice. Of 350 controls who met inclusion criteria, 24 were excluded because they were discharged to hospice (n = 9), transferred to court or law enforcement (n = 2), or died during the index hospitalization (n = 13). This resulted in a final analytic sample of 75 BOLT enrollees and 326 historical controls prior to matching.

Demographic and clinical characteristics of the BOLT enrollees and historical controls are shown in Table 1. Before matching, the groups differed significantly by sex, race, and ethnicity, with the BOLT group having a higher proportion of women, those reporting “other” race, and Hispanic ethnicity. BOLT enrollees also had significantly higher past-month inpatient utilization at the time of the index hospitalization, but significantly lower past-month ED utilization. BOLT enrollees had significantly higher prevalence of opioid use disorder and stimulant use disorder than controls (opioid use disorder: 97% in BOLT enrollees vs 88% in controls [*P* = .017]; stimulant use disorder: 81% in BOLT enrollees vs 70% in controls [*P* = .047]). Alcohol use disorder prevalence was not significantly different. BOLT enrollees and historical controls had a significantly different distribution of the most severe SIRI at index hospitalization (*P* = .015) with BOLT enrollees having lower prevalence of endocarditis (17% in BOLT vs 21% in controls) and bacteremia/unspecified (40% in BOLT vs 52% in controls), higher prevalence of epidural abscess (13% in BOLT vs 6% in controls) and osteomyelitis (13% in BOLT vs 5% in controls), and similar prevalence of septic arthritis (16% in both groups).

**Table 1.** Sample Sociodemographic and Clinical Characteristics Before Matching^a^

	Controls (n = 326)	BOLT (n = 75)	*P* Value^b^
Sociodemographic characteristics^a^			
Age, median (IQR)	42 (35–53)	42 (37–49)	.750
Sex, n (%)			
Male	229 (70)	42 (56)	.017
Female	97 (30)	33 (44)	
Race, n (%)			
White	194 (60)	49 (65)	<.001
Black	85 (26)	6 (8)	
Other	44 (14)	20 (27)	
Missing	3 (1)	0 (0)	
Ethnicity, n (%)			
Non-Hispanic	271 (83)	54 (72)	.019
Hispanic	53 (16)	21 (28)	
Missing	2 (1)	0 (0)	
Insurance category, n (%)			
Medicaid	259 (79)	63 (84)	.562
Medicare	37 (11)	6 (8)	
Other	29 (9)	5 (7)	
Missing	1 (0)	1 (1)	
Clinical characteristics			
Age-adjusted Charlson index, median (IQR)	2 (1–3)	2 (1–3)	.521
Past-month health care utilization^c^			
Inpatient stays, median (IQR)	0 (0–0)	0 (0–1)	.025
Inpatient stays, mean (SD)	0.21 (0.56)	0.33 (0.62)	
ED visits, median (IQR)	0 (0–1)	0 (0–1)	.014
ED visits, mean (SD)	0.80 (1.4)	0.43 (0.79)	
SUD diagnoses,^d^ n (%)			
Opioid use disorder	287 (88)	73 (97)	.017
Stimulant use disorder	228 (70)	61 (81)	.047
Alcohol use disorder	117 (36)	19 (25)	.082
SIRI diagnoses,^e^ n (%)			
Endocarditis, n (%	67 (21)	13 (17)	.015
Epidural abscess, n (%)	20 (6)	10 (13)	
Septic arthritis, n (%)	51 (16)	12 (16)	
Osteomyelitis, n (%)	17 (5)	10 (13)	
Bacteremia/unspecified, n (%)	171 (52)	39 (40)	
Index hospitalization characteristics			
Index length, d, median (IQR)	8 (4–15)	11 (6–17)	.06
Index ICU stay, n (%)	76 (23)	10 (13)	.06
Consult orders			
Addiction consult ordered, n (%)	216 (68)	64 (88)	.001
Infectious disease consult ordered, n (%)	191 (60)	51 (70)	.135
Psychiatry consult ordered, n (%	58 (18)	9 (12)	.219
Surgery consult ordered, n (%)	177 (56)	53 (73)	.009
Addiction medications administered			
Any SUD treatment, n (%)	256 (79)	73 (97)	<.001
Methadone, n (%)	185 (57)	67 (89)	<.001
Buprenorphine, n (%)	78 (24)	11 (15)	.082
Naltrexone, n (%)	6 (2)	0 (0)	.599
Acamprosate, n (%)	9 (3)	1 (1)	.696
Topiramate, n (%)	8 (2)	4 (5)	.249
Discharge medications			
MOUD, n (%)	200 (61)	57 (76)	.017
Polypharmacy, n (%)	176 (54)	49 (65)	.074
Antifungal, n (%)	3 (1)	2 (3)	.236
IV antibiotic, n (%)	44 (14)	13 (17)	.391
Oral antibiotic, n (%)	142 (44)	38 (51)	.264

Abbreviations: BOLT, Boston Healthcare for the Homeless Program Infectious Disease Outreach Liaison Team; ED, emergency department; EHR, electronic health record; IQR, interquartile range; IV, intravenous; MOUD, medication for opioid use disorder; SIRI: serious injection-related infection; SUD, substance use disorder.

^a^Based on EHR data at the time of the patient's index hospitalization.

^b^Calculated excluding missing categories, when relevant.

^c^Pastmonth outcomes refer to the 30 d leading up to the index hospitalization.

^d^SUD diagnoses in the past 90 d.

^e^SIRIs at the index hospitalization for which the patient was hospitalized. Mutually exclusive categories are shown; individuals were assigned to the most severe infection experienced (from most to least severe: endocarditis, epidural abscess, septic arthritis, osteomyelitis, and bacteremia/unspecified).

BOLT enrollees also had significantly higher probability of receiving an addiction consult, surgery consult, and/or methadone during the index hospitalization (BOLT/control: 88%/68% [*P* = .001], 73%/56% [*P* = .00], and 89%/57% [*P* < .001], respectively), but there were no significant differences between groups in infectious disease consults, psychiatry consults, or receipt of buprenorphine, naltrexone, acamprosate, or topiramate during the index hospitalization. Further, while the BOLT group was more likely to receive MOUD as a discharge medication (76% in BOLT, vs 61% of controls, *P* = .017), there were no differences in other discharge medications (polypharmacy, IV antibiotics, oral antibiotics, or antifungals). Last, there were no significant differences in length of index hospitalization stay or whether an ICU visit occurred during the index hospitalization (Table 1).

### Unadjusted Outcomes

Before matching, there were no significant differences in the outcomes of interest between BOLT enrollees and historical controls (Table 2). The mean (SD) 90-day postindex number of hospitalizations was 0.87 (1.4) and 0.78 (1.3) in the BOLT and control groups, respectively (*P* = .830). The mean (SD) number of ED visits was 1.0 (1.7) and 1.4 (2.6) in the BOLT and control groups, respectively (*P* = .393). For BOLT enrollees and historical controls, respectively, the mean (SD) probability of PDD from the index hospitalization was 0.35 (0.48) and 0.28 (0.45) (*P* = .224).

**Table 2.** Unadjusted Inpatient Utilization, ED Utilization, and PDD Outcomes in BOLT Enrollees and Historical Controls

Unadjusted Outcome	Controls (n = 326)	BOLT (n = 75)	*P* Value
90-d inpatient utilization count, mean (SD)	0.78 (1.3)	0.87 (1.4)	.830
90-d inpatient utilization count, median (IQR)	0 (0–1)	0 (0–1)	
90-d ED utilization count, mean (SD)	1.4 (2.6)	1.0 (1.7)	.393
90-d ED utilization count, median (IQR)	0 (0–2)	0 (0–1)	
PDD from index hospitalization, n (%)	90 (28)	26 (35)	.224

Abbreviations: BOLT, Boston Healthcare for the Homeless Program Infectious Disease Outreach Liaison Team; ED, emergency department; IQR, interquartile range; PDD, patient-directed discharge.

### Propensity Score–Matched Analysis

**Figure 1.**
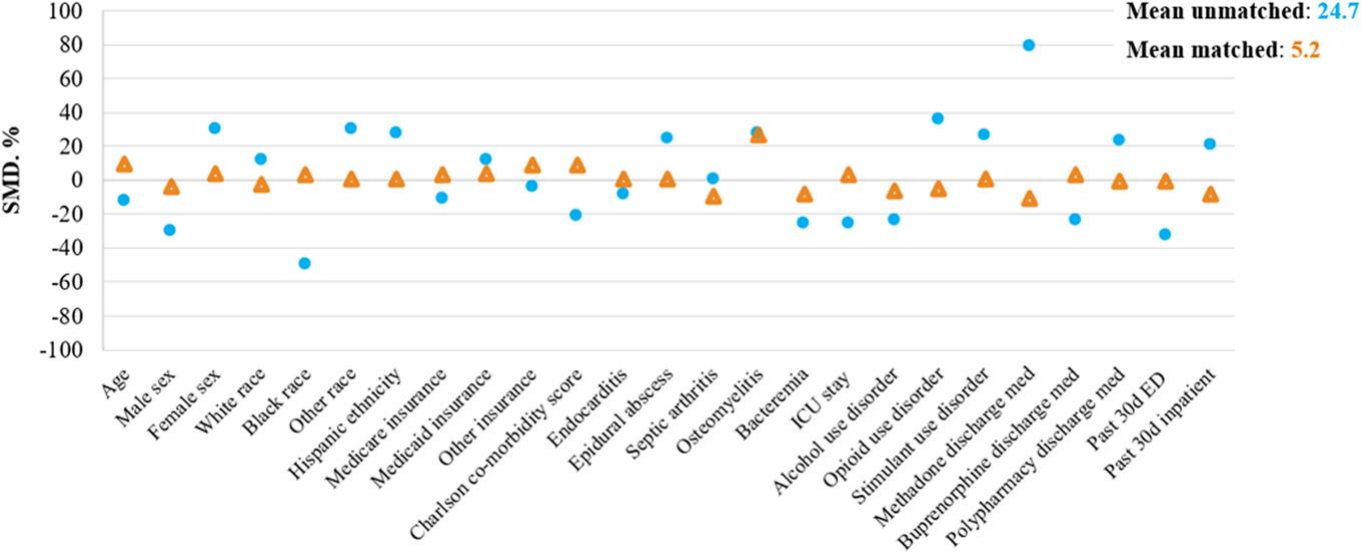
SMD between BOLT enrollees and historical controls, before and after propensity score matching. BOLT, Boston Healthcare for the Homeless Program Infectious Disease Outreach Liaison Team; ED, emergency department; ICU, intensive care unit; SIRI, serious injection-related infection; SMD, standardized mean difference. The figure shows the SMD (expressed as a percentage) in variables used for matching between BOLT enrollees and historical controls, before and after propensity score matching. The included variables are shown on the *x*-axis, and the SMD is shown on the *y*-axis. The dots represent the SMD values before matching, and the triangles represent the values after matching. The SMD values across all variables are shown in the upper right corner of the figure. SMD of <10% was achieved on average overall, and for most variables except for serious injection-related infection type of osteomyelitis and methadone administration during the index hospitalization.

In the matched analysis, 74 BOLT enrollees and 121 historical controls were included. After matching, the overall standardized mean difference (SMD) (expressed as a percentage) between BOLT enrollees and historical controls was 5.2%, which was reduced from 24.7% before matching (Figure 1). Most variables used in matching achieved an absolute SMD of <10%; however, exceptions were osteomyelitis (26.5%) and methadone administration at the index hospitalization (10.9%). Variation in matching parameters did not improve balance, which likely reflects substantial underlying differences in these variables between the groups (Supplementary Table 3).

After matching, there were still no statistically significant differences between the comparison group and BOLT enrollees for any of the primary or secondary outcomes (Table 3). Regression analyses showed that BOLT enrollees and historical controls had nearly identical number of inpatient visits 90 days after the index hospitalization (*P* = .982) and a nonsignificant reduction of 0.35 ED visits compared with matched controls over this period (*P* = .288). Probability of PDD from the index hospitalization was also similar between groups (*P* = .946).

**Table 3.** Regression Results: Difference in Inpatient Utilization, ED Utilization, and PDD Between Propensity Score Matched BOLT Enrollees and Historical Controls

Outcome^a^	Regression Coefficient (95% CI)^b,c^	*P* Value
90-d inpatient utilization, difference in count between BOLT enrollees and historical controls	0.0 (−.42, .43)	.982
90-d ED utilization, difference in count between BOLT enrollees and historical controls	−0.35 (−.99, .30)	.288
Patient-directed discharge, percentage point difference between BOLT enrollees and historical controls	0.5 (−14.2, 15.2)	.946

Abbreviations: BOLT: Boston Healthcare for the Homeless Program Infectious Disease Outreach Liaison Team; ED: emergency department; PDD, patient-directed discharge.

^a^Outcomes shown for the propensity score–matched sample of 74 BOLT enrollees and 121 matched controls.

^b^Individuals were matched on age, Charlson comorbidity score, sex, race, ethnicity, insurance, serious injection-related infection type at index hospitalization, whether index hospitalization was associated with an intensive care unit stay, medication for opioid used disorder administered during hospitalization, past 3-mo substance use disorder diagnoses, past-month ED and inpatient utilization counts, and whether polypharmacy-related medication was prescribed as discharge medication. In a doubly robust approach, these same variables were controlled for in ordinary least squares regression.

^c^In ordinary least squares regression, inpatient and ED utilization coefficients are expressed as a change in the number of visits, and PDD coefficients are expressed as changes in probability in percentage points.

Conclusions did not change across the sensitivity analyses varying matching approaches, regression specifications, or outcome definitions (Supplementary Table 3).

## DISCUSSION

In this analysis, we did not find statistically significant associations between enrollment in BOLT and 90-day re-hospitalizations and ED visits or in PDD among people with housing instability and SUD hospitalized for SIRI. For PDD and inpatient hospitalization, the differences between BOLT enrollees and controls were very close to zero. For ED visits, BOLT enrollees had lower utilization after their index hospitalization, but this difference was not statistically significant.

Prior literature has demonstrated that outpatient supports can be beneficial for PWID hospitalized with SIRIs. For example, a study conducted across 3 sites in Missouri found that PWID hospitalized with SIRI who left via PDD and were prescribed oral antibiotics had similar re-hospitalization rates as those who remained in the hospital to complete IV antibiotics; however, they required intensive outpatient supports to successfully complete oral antibiotics [11]. While we did not find a significant association between BOLT enrollment and re-hospitalization, ED visits, or PDD, this prior evidence highlights that BOLT may have supported patients in other ways not captured by our outcomes. For example, our data do not include more proximal intermediate outcomes in either the BOLT or comparison groups, including antibiotic completion, mental health outcomes (eg, psychiatric treatment, social connectedness) or linkage to resources such as MOUD, HIV and hHepatitis C virus treatment or prevention services, housing, or case management. While healthcare utilization is an important outcome with clear resource allocation implications for payers and funders, it is also quite distal. Therefore, as suggested in other evaluations of supportive programs [20], researchers may need to consider tempering expectations regarding impacts on utilization and instead prioritize these more proximal outcomes. However, it is also notable that during the study, the acceptability of antibiotic use for treatment of SIRIs among providers in the field of infectious disease was increasing [8]. Somewhat higher oral antibiotic use in the BOLT versus comparison group may reflect this trend, though the difference is nonsignificant (which could reflect lower infection severity in the comparison group). Nonetheless, it is reassuring that amidst increasing use of oral antibiotics for treatment of SIRIs that rates of these outcomes remained similar, suggesting that this intervention did not worsen acute care utilization.

Our null findings may reflect several factors. First, this analysis evaluated the first 15 months of the program, when adaptations were still frequently occurring, and there were staffing-related challenges, including a nursing shortage. Prior literature suggests that in general, programs tend to improve their implementation strategies over time, increasing their effectiveness [21]. Second, also because we only had 15 months of data, we had a small treated sample, reducing our power to detect small differences between groups. This is particularly relevant for the ED outcome, which showed a nonsignificant decrease of 0.35 ED visits in the 90-day post period (95% CI: −.99 to .30), whereas the inpatient and PDD outcomes were nearly zero. Future research on the BOLT program would benefit from a larger sample with more follow-up time (eg, including more years of data) and a robust implementation evaluation. Notably, qualitative evaluation of stakeholder and patient perspectives on their experience with BOLT would be essential for interpreting our quantitative findings. Here, we were not able to conduct these larger-scale analyses due to lack of resources for data acquisition and analyst time. While BOLT itself was funded as an operational initiative, a prospective evaluation was not specifically funded. Given that deeper understanding of BOLT implementation and effectiveness is key to its long-term sustainability and quality improvement, this highlights the importance of funding program evaluation alongside implementation efforts [22].

This analysis has several limitations. As mentioned, we were unable to differentiate between implementation and effectiveness factors, and our small sample limited power to detect statistically significant differences in outcomes. Additionally, while we used a propensity score matching approach to balance observed differences between BOLT enrollees and historical comparisons, we were unable to achieve optimal matching; in particular, BOLT enrollees had a higher prevalence of osteomyelitis, which remained even after matching, likely reflecting underlying persistent differences between the groups, with BOLT enrollees potentially having more chronic, re-occurring conditions that could be associated with differential healthcare utilization over time. If there were also key unobserved differences between the BOLT and comparison groups, our estimates could be biased, despite use of a doubly-robust model. Further, because we only had access to electronic health records (and not all-payer claims), we were not able to observe utilization that occurred outside of BMC, including utilization at Boston Health Care for the Homeless Program; if this type of utilization was different between BOLT and control groups, results could be biased. Similarly, if BOLT patients were transferred from an external hospital, they were excluded from the analysis (this was only the case for <5 patients). The results also may have been affected by time-related bias given the use of historical (vs contemporaneous) controls. Notably, while we excluded the months of the coronavirus disease 2019 pandemic in which hospital utilization was most impacted (March through May 2020), the pandemic may have differentially impacted utilization in the historical control group. Other factors, like expansion of harm reduction resources (eg, naloxone), changes in standards of care (eg, increasing acceptance of oral antibiotic use), or changes in local policies (eg, housing/homelessness policy) could have affected differences over time. Last, some BOLT enrollees had infections that are not typically coded as SIRIs but needed comparable antibiotic adherence support (eg, recurring soft tissue infections). Because we could not reliably identify individuals with these infections at this severity level in the control group, we placed these enrollees in the bacteremia/other category for clinically relevant matching and to preserve sample size, but this may have also contributed to underlying differences between groups.

In conclusion, we did not find significant associations between the BOLT intervention and the outcomes we evaluated: PDD and 90-day re-hospitalization and ED utilization. These findings point to the need for more research to understand BOLT implementation, including barriers, facilitators, changes over time, cost-effectiveness, as well as the importance of assessing more proximal outcomes in comparative analyses. This paper has implications for future research on similar interventions and other types of operational initiatives that are implemented without robust evaluation infrastructure. BOLT would benefit from additional resources to support comprehensive monitoring and evaluation of implementation factors and short- and long-term outcomes.

## Supplementary Material

ofag481_Supplementary_Data

## Supplementary Data

Supplementary materials are available at *Open Forum Infectious Diseases* online. Consisting of data provided by the authors to benefit the reader, the posted materials are not copyedited and are the sole responsibility of the authors, so questions or comments should be addressed to the corresponding author.
