# Supplementary material for: Outcomes of a Program to Support Outpatient Antibiotic Adherence Among People Experiencing Housing Instability With Serious Injection Drug-Related Infections in Boston, MA: A Propensity Score Matched Study
Source: Open Forum Infect Dis. 2026 Aug 5;13(8):ofag481. doi: 10.1093/ofid/ofag481 (PMC13458145; doi:10.1093/ofid/ofag481)
Supplement: Supplementary file 1 [file ofag481_supplementary_data.docx]

**Supplemental appendix:**

**Outcomes of a program to support outpatient antibiotic adherence among people experiencing housing instability with serious injection drug-related infections in Boston, MA: a propensity score matched study**

Nicole C. McCann,^1^ Ghulam Karim Khan,^2^ Elizabeth Glaser,^2^ Jake R. Morgan,^1^

Dominic Hodgkin,^3^ Simeon D. Kimmel^4,5,6^

**Table of contents.**

| **Supplemental Table 1**. ICD-10 codes used to identify SIRI and SUD | Page 2 | |
| --- | --- | --- |
| **Supplemental Table 2.** List of included discharge medications | Pages 3-4 | |
| **Supplemental Table 3**. Sensitivity analysis results | Page 5 | |
|  |  |  |
|  |  |  |

**Supplemental Table 1. ICD-10 codes used to identify SIRI and SUD**

| **SIRI** | **ICD-10 codes** |
| --- | --- |
| *Endocarditis* | B37.6; I33.0; I33.9; I38; I39 |
| *Osteomyelitis* | M46.2x; M86x |
| *Septic arthritis* | M00.0x; M00.8x; M00.9 |
| *Epidural abscess* | 606.0; G06.1; G06.2 |
| *Bacteremia* | A41.0x; A41.1; A41.2; A41.3; A41.4; A41.5X ; A41.81; A49.9; R78.81; B37.7; B37.9; A40.0; A40.1; A40.3; A40.8; A40.9 |
| **SUD** |  |
| Alcohol-related disorders | F10x |
| Opioid-related disorders | F11x |
| Stimulant disorders | F14x; F15x |

**Abbreviations: ICD-10:** International Classification of Diseases, 10^th^ edition; **SIRI:** serious injection-related infection; **SUD**: substance use disorder

**Supplemental Table 2. List of included discharge medications**

|  | **Medication name** |
| --- | --- |
| *Oral antibiotics* | - Cefpodoxime - Cefadroxil - Levofloxacin - Moxifloxacin - Viprofloxacin - Amoxicillin-clavulanate - Amoxicillin - Penicillin v potassium - Doxycycline - Sulfamethoxazole-trimethoprim - Rifabutin - Rifampin - Tedizolid - Linezolid - Metronidazole |
| *IV antibiotics* | - Ertapenem - Cefazolin - Ceftriaxone - Cefepime - Penicillin - Nafcillin - Ampicillin-sulbactam - DAPTOmycin - Vancomycin - Sodium chloride |
| *Antifungals* | - Fluconazole - Isavuconazonium - Voriconazole |

**Supplemental Table 2. List of included discharge medications, cont.**

| **Medication category** | **Medication name** | |
| --- | --- | --- |
| *Polypharmacy-related* | - Morphine - Ozycodone - Hydropmorphone - Alprazolam - Lorazepam - Diazepam - Clonazepam - Quetiapine - Clonidine - Prochloroperazine - Promethazine - Gabapentin - Prazosin - Pregabalin - Buspirone - Lamotrigine - Valproic acid - Oxycarbazepine - Zolpidem - Trazodone - Pregabalin - Lithium - Divalproez | - Doxepin - Lamotrigine - Fluoxetine - Mirtazapine - Venlafazine - Paroxetine - Amitriptyline - Venlafaxine - Aripiprazole - Olanzapine - Risperidone - Haloperidol - Aripiprazole - Chloropromazine - Ziprasidone - Cariprazine - Lurasidone - Dextroamphetamine-amphetamine - Methylphenidate HCL - Lisdexamfetamine - Dextroamphetamine sulfate - Methylphenidate - Atomoxetine |
| *MOUD* | - Methadone - Buprenorphine-naloxone - Naltrexone | |

**Supplemental Table 3. Sensitivity analysis results**

|  | **Matched, controls, N** | **Matched, BOLT, N** | **SMD, %** | **Regression coefficient**  **(p-value)** | | |
| --- | --- | --- | --- | --- | --- | --- |
|  |  |  |  | ***Inpatient*** | ***ED*** | ***PDD*** |
| Base case^1^ | 121 | 74 | 5.2 | 0.00 (0.982) | -0.35 (0.288) | 0.5 (0.946) |
| *Varied matching approach^2,3^* | | | | | | |
| Inverse probability weighting | 121 | 74 | 5.2 | -0.04 (0.784) | -0.34 (0.204) | 10.0 (0.184) |
| 1:2 matching | 97 | 74 | 5.6 | 0.0 (0.943) | -0.53 (0.163) | -1.3 (0.871) |
| 1:1 matching | 59 | 74 | 9.9 | 0.0 (0.881) | -0.40 (0.335) | 2.4 (0.787) |
| 1:1 matching, no replacement | 69 | 69 | 7.7 | 0.0 (0.960) | -0.36 (0.352) | 2.0 (0.815) |
| 0.1 caliper | 121 | 74 | 4.9 | 0.0 (0.965) | -0.36 (0.274) | 0.0 (0.998) |
| 0.3 caliper | 121 | 74 | 5.2 | 0.0 (0.982) | -0.35 (0.288) | 0.0 (0.946) |
| No common support imposed | 121 | 75 | 5.5 | 0.0 (0.965) | -0.34 (0.293) | 0.2 (0.983) |
| *Varied regression specification^3,4^* | | | | | | |
| Negative binomial regression | 121 | 74 | 5.2 | -0.01 (0.954) | -0.23 (0.354) | NA |
| Logistic regression | 121 | 74 | 5.2 | NA | NA | -0.02 (0.954)^2^ |
| *Varied utilization outcome definition^3^* | | | | | | |
| Binary utilization outcomes | 121 | 74 | 5.2 | -4.0 (0.851)^3^ | -1.7 (0.824)^3^ | NA |

**Abbreviations: BOLT:** Boston Healthcare for the Homeless Program Infectious Disease Outreach Liaison Team; **SMD**: standardized mean difference; **PDD**: patient-directed discharge; **CI:** confidence interval; **ED**: emergency department

^1^Base case parameters were 1:3 matching using a greedy nearest neighbor approach, matching with replacement, with a caliper of 0.2, imposing common support trimming

^2^Varied matching approach maintains base case regression specification (ordinary least squares), where inpatient and ED utilization coefficients are expressed as a change in the number of visits, and PDD coefficients are expressed as changes in probability in percentage points

^3^Inpatient and ED utilization are defined as visit counts in the 90 days after index hospitalization discharge for all instances, except for “binary utilization outcomes” where coefficients represent changes in probability of any visit in percentage points

^4^Negative binomial regression coefficients represent log change in utilization counts, and logistic regression coefficients represent change in log-odds of PDD
